# Performance and outcomes of transvenous rotational lead extraction: Results from a prospective, monitored, global clinical study—“An evolution in extraction”

**DOI:** 10.1016/j.hroo.2021.03.006

**Published:** 2021-03-19

**Authors:** Kunal Shah, Travis Pollema, Ulrika Birgersdotter-Green

**Affiliations:** ∗ Division of Cardiology, Cardiac Electrophysiology Program, Sulpizio Family Cardiovascular Center, La Jolla, California, and the University of California Medical Center, San Diego, California; † Division of Cardiothoracic surgery, Sulpizio Family Cardiovascular Center, La Jolla, California, and the University of California Medical Center, San Diego, California

More than 1 million new cardiovascular implantable electronic devices are inserted yearly across the globe.^1^ Subsequently, lead management is becoming an increasingly vital component of patient care. The guidelines for when a lead should be extracted are well defined.^2^^,^^3^ However, providing safe and effective transvenous lead extractions (TLE), especially in older leads, can be challenging. Operator experience, extraction equipment, and center workflow all contribute to outcomes. Although there is no “gold-standard” extraction technique, both laser cutting sheaths and mechanical rotational sheaths have been used with success.^4^^,^^5^ Most data supporting these methods are derived from large retrospective cohorts and registries. More prospective studies are needed that have the potential both to guide decision-making for extraction centers and to identify long-term patient outcomes.

In this issue of *Heart Rhythm O*^*2*^, Sharma and colleagues^6^ provide an insightful prospective study aimed at understanding the safety and efficacy of the Cook Medical Evolution® RL mechanical rotational TLE device (RELEASE study). Ten medical centers in the United States and Europe were involved, with 230 patients enrolled for a total of 460 leads extracted. All patients underwent a 4-week follow-up visit. Data were verified by an independent clinical monitoring service and all complications were reviewed and adjudicated by an independent clinical events committee. The authors report a high procedural success rate of 96.3% and an impressive median extraction time of 10 minutes per procedure, with median lead implant time of 7.4 years. This time is faster than some other large registry studies, such as ELECTRa, which reports a median extraction time of 19 minutes.^5^ The authors also report a major complication rate of 5.7% (3.0% during extraction, 2.6% on postoperative day 1), which may be higher than comparative studies. They attribute this slightly higher rate to rigorous clinical data gathering and independent clinical events committee review. Of note, this study did not have any isolated superior vena cava injuries or procedural mortality.

In 2010, Hussein and colleagues^7^ reported initial experience with the first-generation mechanical Evolution sheath. This introductory study of 29 patients (41 leads) reported procedural success of 86% with no major complications. In the following decade, multiple studies confirmed the safety and efficacy of this form of mechanical extraction and included the second-generation Evolution RL sheath.^8^ The PROMET study was the largest of this series and included more than 2000 patients. This retrospective, multicenter European study reported complete lead extraction in 96.5% of patients with a 1% occurrence of major complications.^8^ Mazzone and colleagues^9^ presented prospective data on 124 consecutive patients performing extraction exclusively with the Evolution RL in Italy. They had extraction success in 91.6% of patients and no major complications.

This current study by Sharma and colleagues^6^ compares favorably with these studies and includes clinical follow-up data at 4 weeks. It represents one of the largest international, prospective studies of mechanical TLE. The high procedural success rate of 96% and relatively fast extraction times further support mechanical extraction as a viable option for lead management. In addition, this study also adds to the literature because of the rigorous clinical follow-up and independent review. The modestly higher complication rates may be more representative of “real-world” patients, and this study also sheds light on what happens to extraction patients after they leave the hospital.

Sharma and colleagues present compelling data on the use of mechanical rotational TLE techniques, but there are important factors to consider when interpreting the findings. To begin with, the RELEASE study’s impact is somewhat limited by its design as an observational study with no control group or direct comparison. Operators’ intention to treat may have introduced selection bias in the study population, which may not represent all patients who need TLE. The desire to represent “real-world” patients may be hindered by the fact that most extractions were done at high-volume centers (9 of 10) located in the United States and Europe only. Furthermore, this study reports an infectious indication for extraction in only 38.5% of patients. Although they do attempt to address this issue, most studies report a 45%–50% infectious indication.^8^^,^^9^ Extraction for infected cardiovascular implantable electronic devices has consistently been shown to lead to higher in-hospital and 30-day mortality.^10^ The lower number of device infections in this study may also skew the overall complication rate.

Overall, the RELEASE study is a well-executed and rigorous study of a mechanical rotational TLE tool (Cook Medical Evolution RL). It will add to the literature and provide insight on realistic clinical expectations and outcomes for extraction patients. Additionally, it highlights the need for more prospective studies of lead extraction and provides a roadmap for success. TLE will continue to be an important but often challenging procedure for patients. Although this study analyzed the use of mechanical extraction, it is also important to have multiple tools available, such as laser cutting sheaths and femoral snares, for every case. Each extraction presents unique challenges, and occasionally, a combination of techniques and tools is needed to safely remove each lead. Ultimately, operator and center expertise combined with the right equipment will lead to the best outcomes.

## Funding Sources

This research did not receive any specific grant from funding agencies in the public, commercial, or not-for-profit sectors.

## Disclosures

The authors have no conflicts to disclose.

## Authorship

All authors attest they meet the current ICMJE criteria for authorship.
